# The next generation of metastatic melanoma: uncovering the genetic variants for anti-BRAF therapy response

**DOI:** 10.18632/oncotarget.7175

**Published:** 2016-02-03

**Authors:** Rosamaria Pinto, Simona De Summa, Sabino Strippoli, Brunella Pilato, Amalia Azzariti, Gabriella Guida, Michele Guida, Stefania Tommasi

**Affiliations:** ^1^ IRCCS Istituto Tumori “Giovanni Paolo II”, Molecular Genetics Laboratory, Bari, Italy; ^2^ IRCCS Istituto Tumori “Giovanni Paolo II”, Oncology Unit, Bari, Italy; ^3^ IRCCS Istituto Tumori “Giovanni Paolo II”, Clinical and Preclinical Pharmacology Laboratory, Bari, Italy; ^4^ University of Bari, Department of Medical Biochemistry, Bari, Italy

**Keywords:** metastatic melanoma, next generation sequencing, BRAF inhibitors, progression, Ion Torrent

## Abstract

Metastatic melanoma (MM) is a highly aggressive cancer with a median overall survival of 6–9 months, notwithstanding the numerous efforts in development of new therapeutic approaches. To this aim we tested the clinical applicability of the Ion Torrent Personal Genome Machine to simultaneously screen MM patients in order to individuate new or already known SNPs and mutations able to predict the duration of response to BRAF inhibitors. An Ampliseq Custom Panel, including 11 crucial full length genes involved in melanoma carcinogenesis and therapy response pathways, was created and used to analyze 25 MM patients. We reported BRAF^V600^ and NRAS^Q61^ mutations in 68% and 24% of samples, respectively. Moreover, we more frequently identified the following alterations related to BRAF status: PIK3CA^I391M^ (44%) and KIT^D737N^ (36%) mutations, CTLA4^T17A^ (52%), MC1R^V60L^ (32%) and MITF^S473A^ (60%) polymorphisms. Considering the progression free survival (PFS), statistical analyses showed that BRAF^V600^ patients without any of these more frequent alterations had a higher median PFS. Protein structure changes seem to be due to these variants by *in silico* analysis. In conclusion, a Next-Generation Sequencing approach with custom panel may provide new information to evaluate tumor-specific therapeutic susceptibility and individual prognosis to improve the care of MM patients.

## INTRODUCTION

BRAF mutations are present in about 50% of melanomas, causing an over-activation of the MAPK/ERK pathway involved in cell proliferation and survival. The most frequent mutation (90% of cases) results in a substitution of a valine in glutamic acid at amino acid 600 (BRAF^V600E^). In August 2011, the FDA, approved Vemurafenib (Zelboraf; Roche) for treatment of BRAF^V600E^ metastatic melanoma due to improved objective response, progression free survival and overall survival showed in several clinical trial [1]. Inhibition of mutated BRAF and consequently reduction of ERK phosphorylation leads to suppression of cyclin D1, induction of expression of the cell-cycle inhibitor p27, and, eventually, to cell-cycle arrest. Unfortunately, responses to BRAF inhibitors are short-lived, with evidence of disease progression within 6–8 months after the beginning of therapy due to the recovery of MAPK signaling or activation of alternative signaling pathways, such as PI3K/AKT/mTOR by IGF-1R or PDGFRb up-regulation [2]. Mutational activation of NRAS is, instead, a common mechanism of resistance to BRAF inhibitors due to increased formation of RAF dimers, against which the drug cannot work [3]. Furthermore, in cells with mutated NRAS, BRAF inhibitors may induce paradoxical activation of the downstream factor MEK1. Another proposed resistance mechanism to BRAF inhibitors is represented by secondary mutations of MEK1 that may also result in reactivation of the MAPK pathway and cause resistance to BRAF inhibitors [3].

An established strategy to overcome BRAF inhibitor resistance is the combination of BRAF inhibitor with MEK inhibitor that targets another protein in the MAPK pathway as demonstrated in recent clinical trials [4].

Moreover, a latest approach is represented by the combination of immunotherapy and targeted therapy, trying to overcome the great toxicity caused by this combination [5–7].

However, although several studies on genetic alterations have been conducted, the molecular mechanisms underlying this very small range of response time to BRAF inhibitors are to date unknown.

In recent years, Next-Generation Sequencing (NGS) platforms, also known as massive parallel sequencing, have drastically decreased the time and cost associated with a comprehensive cancer genome analysis [8–14]. This sequencing technique allowing whole-genome, whole-exome sequencing but also the screening of specific gene mutations, provides highly relevant advances in a clinical setting since a comprehensive mutational screening of tumors could be useful for the best therapeutic assessment [15, 16]. The sensitivity of NGS is higher than traditional methods such as Sanger sequencing (detection of 2–10% versus 15–25% allele frequency). Furthermore, NGS technologies facilitate the screening of multiple genes with limited starting material derived from blood or FFPE tissues, differently to Sanger's sequencing method that requires relatively large DNA quantities to assess single gene alterations.

In this study we tested the clinical applicability of the NGS platform Ion Torrent Personal Genome Machine (Life Technologies, Carlsbad, CA), to simultaneously screen metastatic melanoma patients in order to individuate new or already known SNPs and mutations which could be related with different response duration to BRAF inhibitors. We created an Ampliseq Custom Panel (Life Technologies, Carlsbad, CA) including 11 crucial full length genes involved in melanoma carcinogenesis and therapy response pathways.

## RESULTS

### Alteration frequencies and sensitivity detection of NGS variant calling

**Figure 1:**
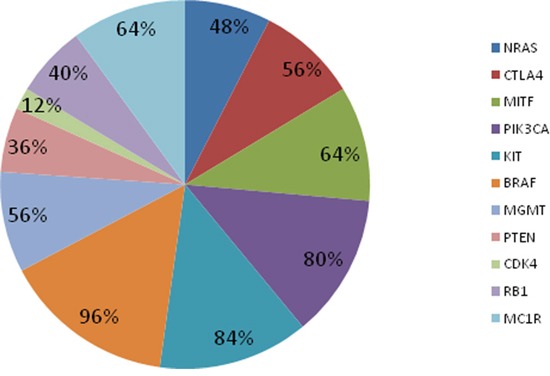
The variant frequency for each gene of the custom panel MITF, PIK3CA, KIT, CTLA4, BRAF, MGMT and MC1R resulted the more frequently altered genes.

All 25 amplified samples showed at least one alteration in at least one of the 11 melanoma cancer-related genes (Figure 1). Querying CLINVAR, 12 patients presented alterations in NRAS and only 7 of these have alterations already evidenced as pathogenic in cancer; 14 patients presented alterations in CTLA4 and 13 of these have alterations already evidenced as a risk factor in pathologies other than cancer; 20 patients showed PIK3CA alterations and 21 patients presented alterations in KIT but none seems to be pathogenic; all patients but one presented alterations in BRAF and 17 have a mutation in codon 600; 12 patients presented alterations in RB1 and 1 of these has alterations already evidenced as pathogenic in retinoblastoma; 16 patients presented alterations in MC1R and 15 of these have alterations already evidenced as associated to pigmentation disorders; furthermore, 16, 9, 14 and 3 patients presented no previously reported alterations in MITF, PTEN, MGMT and CDK4 respectively (see Suppl. Table 1).

We reported BRAF^V600^ and NRAS^Q61^ mutations in 17/25 (68%) and 6/25 (24%) samples, respectively. Moreover we more frequently identified the following alterations: PIK3CA^I391M^ (44%) and KIT^D737N^ (36%) mutations, CTLA4^T17A^ (52%), MC1R^V60L^ (32%) and MITF^S473A^ (60%) polymorphisms.

Ten nanograms of DNA were processed according to the manufacturers' protocol. In 25/31 (81%) samples, an adequate library for subsequent sequencing was obtained. No library amplification was observed in 6 FFPE samples probably due to the low quality of the extracted DNA. The sensitivity of our experimental setup was tested analyzing one FFPE sample for the frequent mutation BRAF^V600E^ (c.1799 T>A) and for the polymorphism CTLA4^T17A^ (c.49 A>G). DNA dilution 1:30 corresponding to 3% of tumor DNA content was investigated to verify the ability of Ion Torrent calling. The sample as it was and its dilution showed identical alterations (data not shown).

To verify the variant calling accuracy, BRAF and NRAS mutations were also analyzed by Sanger sequencing and Real-Time PCR ARMS methods respectively. BRAF^V600^ was detected in 16 samples by both methods, and 1 was missed by Sanger sequencing. NRAS^Q61^ was detected in 4 samples by both methods and 2 were missed by the Real-Time PCR ARMS method. Moreover concordance between Ion Torrent, Sanger and ARMS methods was present in all cases without mutations in BRAF or NRAS. Generally, BRAF and NRAS mutations were mutually exclusive, even if one case of our sample set showed both mutations.

### BRAF mutation and risk of concomitant multiple alterations

A generalized linear model analysis considering all alterations detected in all genes included in our Ampliseq custom panel was applied to study the risk of BRAF mutation when other genes resulted altered. Only CTLA4 and KIT resulted significantly associated to BRAF status. In particular, we found that if CTLA4 was altered, the risk of a concomitant BRAF^V600^ mutation was low, on the contrary this risk was high if KIT was altered [OR, 0.1-95%CI: 0.05%0.74; OR, 12.5- 95%CI: 1.82%125.77 respectively]. These results were confirmed by the Chi-square test evidencing that 59% of patients with BRAF^V600^ were wild type for CTLA4 (p=0.08), while 88% of patients had KIT altered and BRAF^V600^ (p=0.03).

Considering the more frequent variations above reported, our linear model analysis revealed that a BRAF^V600^ patient could have a low risk of presenting also CTLA4^T17A^, MITF^S473A^ or MC1R^V60L^ alteration and PIK3CA^I391M^ mutation [OR: 0.077- 95%CI: 0.003%0.58; OR: 0.11- 95%CI: 0.005%0.83; OR:0.51- 95%CI: 0.08%3.37; OR: 0.13- 95%CI: 0.015%0.83 respectively]. The Chi-square test evidenced that 65% of BRAF^V600^ patients lacked CTLA4^T17A^ (p=0.04). On the contrary, the mutation KITD737N resulted associated to BRAFV600 [OR: 2.1-95%CI: 0.35%17.33].

### Genetic pattern related to clinical outcome

A subset of 17 BRAF mutated patients, treated with Vemurafenib and Dabrafenib, was followed up until progression. Progression free survival (PFS) was considered with respect to alterations in CTLA4, MITF, PIK3CA, KIT and MC1R by Kaplan–Meier curves and log-rank analysis.

Considering the presence of any alteration in CTLA4 and KIT genes, statistical analysis evidenced that patients without variations had a higher median PFS (4 vs 7 and 7 vs 21, respectively). Interestingly, when we considered the most frequent alteration (PIK3CA^I391M^, KIT^D737N^, CTLA4^T17A^, MC1R^V60L^ and MITF^S473A^), analyses showed that BRAF^V600^ patients without any of these had a higher median PFS, even if the statistical significance was not reached probably due to the small number of patients analyzed.

The Cox proportional hazards regression model was used to determine the risk that the identified alterations could lead to a progression event (Table 1). The univariate Cox hazard regression model demonstrated a possible role for altered KIT and CTLA4 and for CTLA4^T17A^, MC1R^V60L^ and MITF^S473A^ as risk factors. Multivariate analysis that considered all CTLA4 and KIT alterations or the most frequent alteration for each gene with respect to gender, age and stage evidenced all single alterations as a risk marker of progression. In particular MC1R^V60L^ results significantly associated with melanoma progression (p=0.004).

**Table 1:** Univariate and multivariate analyses through the COX regression model

	UNIVARIATE		MULTIVARIATE	
	HR	95% CI	HR	95% CI
*CTLA4_any alteration*	2.285	0.81÷6.39	2.02	0.54÷7.51
*KIT_any alteration*	2.402	0.31÷18.38	1.15	0.11÷11.80
*CTLA4^T17A^*	1.917	0.67÷5.43	1.46	0.39÷5.45
*PIK3CA^I391M^*	0.67	0.21÷2.13	2.77	0.52÷14.63
*MITF^S473A^*	1.91	0.70÷5.54	2.54	0.40÷15.90
*KIT^D737N^*	0.94	0.33÷2.66	1.69	0.45÷6.25
*MC1R^V60L^*	2.735	0.81÷9.18	14.43*	2.35÷88.49

(*) The analyses considered any CTLA4 or KIT alterations or the single reported more frequent alterations with respect to gender, age and stage. The significant results (p<0.05) are indicated

**Figure 2:**
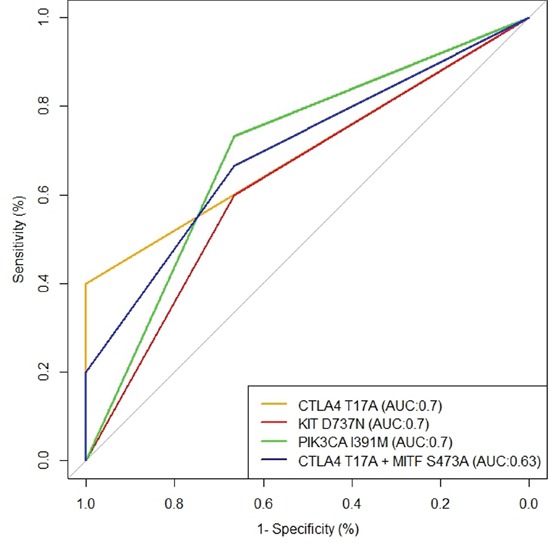
The best ROC curves evaluated to exploit the role of the five alterations more frequently found in relation to anti-BRAF therapy response The best accuracy in discriminating short and long PFS was reached by CTLA4^T17A^, PIK3CA^I391M^ and KIT^D737N^ (AUC:0.7). CTLA4^T17A^ when simultaneously present with MITF^S473A^ showed a lower accuracy in predicting disease progression (AUC:0.63).

ROC curves and the AUC (Area Under Curve) parameter were evaluated to exploit the role of the five alterations more frequently found, in predicting duration of response to anti-BRAF therapy. In particular, PFS was taken into account considering alterations both as individually and as concomitantly present in patients. In Figure 2, best curves are represented. In particular, the best accuracy in discriminating short and long PFS was reached by CTLA4^T17A^, PIK3CA^I391M^ and KIT^D737N^ (AUC: 0.7). CTLA4^T17A^, when simultaneously present with MITF^S473A^, showed a lower accuracy in predicting disease progression (AUC: 0.63).

### Prediction and structural analysis of the most frequent alterations

To assess the hypothetical pathogenic role of the above alterations (CTLA4^T17A^, MITF^S473A^, PIK3CA^I391M^, MC1R^V60L^ and KIT^D737N^), we used some bioinformatic tools (SIFT, Polyphen-2, CONDEL, SNP&GO and PANTHER). The PIK3CA^I391M^ and KIT^D737N^ were predicted to be deleterious with 3/5 tools, while the CTLA4^T17A^ and MITF^S473A^ were considered benign. MC1R^V60L^ was considered damaging only by Polyphen-2.

**Figure 3:**
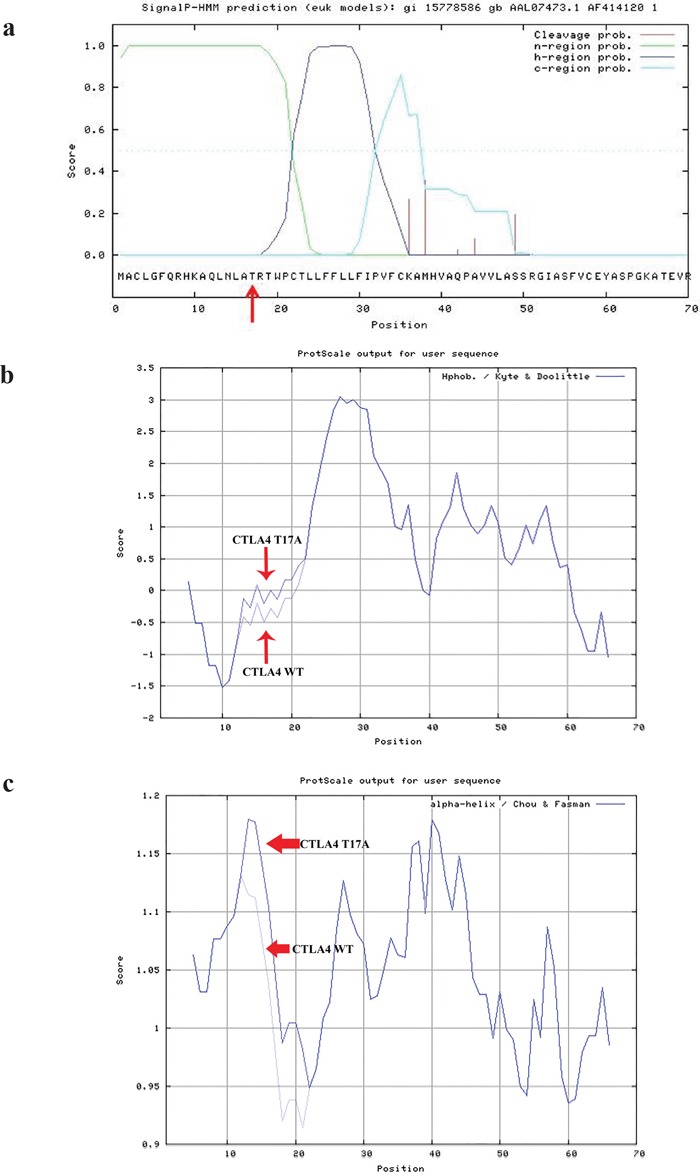
CTLA4 structural analysis The CTLA4^T17A^ variation prediction analysis has been performed through SignalIP 3.0 (http://www.cbs.dtu.dk/services/SignalP-3.0/) **a.** This variant is located in the n-region which interacts with the translocation machinery and with the negatively charged phospholipids in the lipidic bilayer of the membrane a. The T17A signal peptide showed higher hydrophobicity, measured through the Kyte and Doolittle scale than the wild-type **b**. A higher propensity to form an α-helix in the Chou and Fasman scale, due to the variant CTLA4^T17A^, was shown **c.**

Moreover to truly clarify the biological characteristic of the 5 alterations, we performed a structural analysis in silico. Codon 17 in the CTLA4 gene is located in the signal peptide, whose secondary structure is important for its functionality. SignalIP indicated positions 37-38 to be the most likely cleavage site. Figure 3a shows that the Thr17 is located in the n-region, which interacts with the translocation machinery and with the negatively charged phospholipids in the lipidic bilayer of the membrane. The T17A signal peptide showed higher hydrophobicity, measured through the Kyte and Doolittle scale, than the wild-type (Figure 3b). Moreover, we observed a higher propensity to form α-helix in the Chou and Fasman scale for the altered signal sequence (Figure 3c).

**Figure 4:**
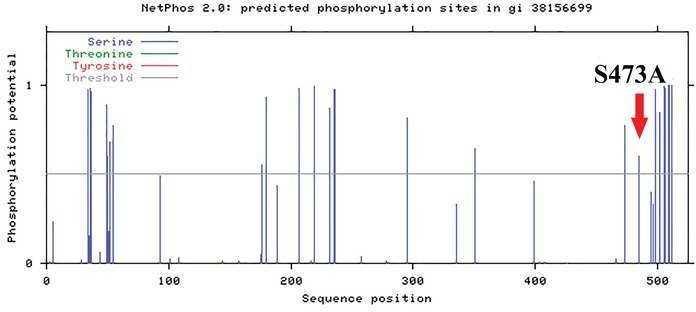
MITF structural analysis MITF^S473^ is included in the 25 serines which could be potential phosphorylation sites. This analysis has been conducted through NetPhos 2.0.

The MITF gene alteration frequently identified in our study is S473A. The importance of phosphorylation for the functionality of this protein in different conditions is well known. NetPhos 2.0 results indicated that MITF has 25 serines which could be potential phosphorylation sites. Interestingly, Ser473 (Figure 4) is included in the prediction, suggesting a potential influence of S473A alteration in the function of MITF.

**Figure 5:**
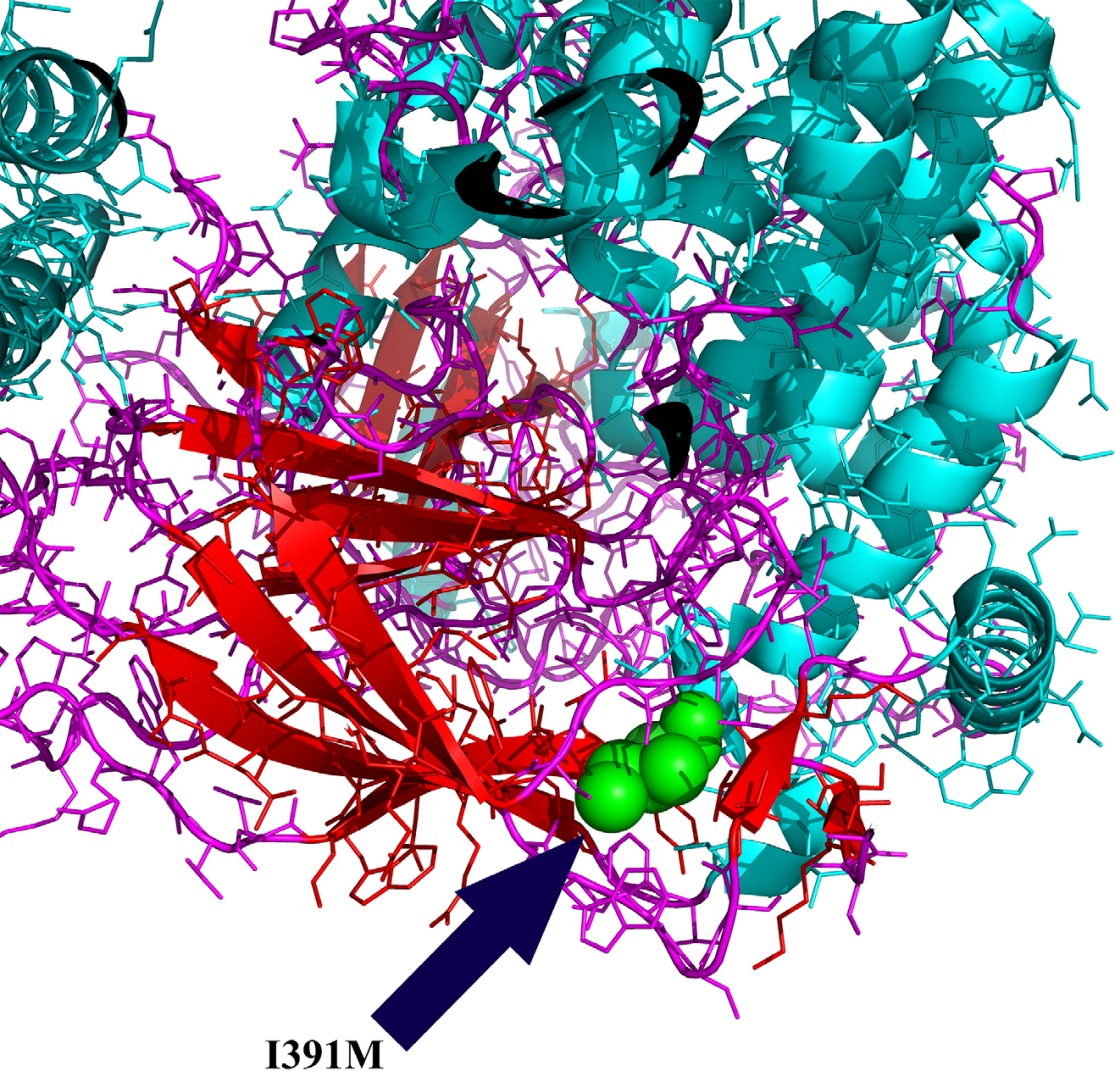
PIK3CA structural analysis PIK3CA^I391^ (residue in green) is located in the C2 domain, responsible for phospholipid membrane binding. I391 resulted the only residue to work as a loop between the core structure of the C2 domain and an α-helix. The analysis has been conducted through the PyMOL Molecular Graphics System (Version 1.4.1 Schrödinger, LLC).

**Figure 6:**
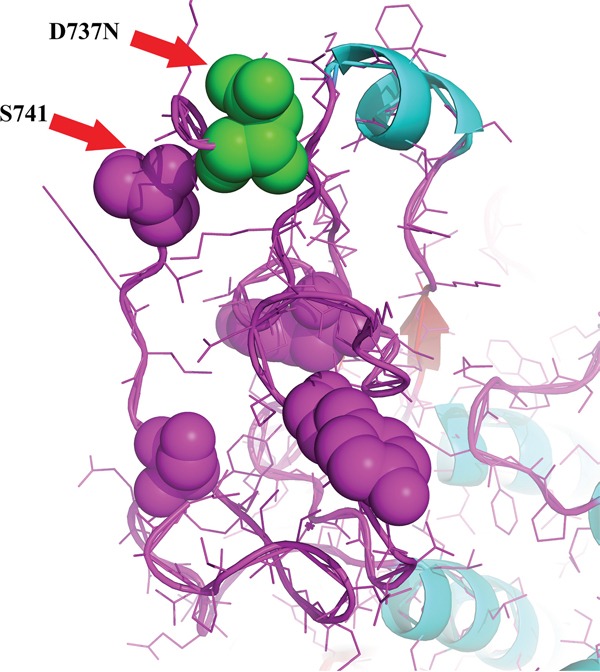
KIT structural analysis KIT^D737^ (residue in green) is located in a loop region near to catalytic tyrosine and serine residues, in particular to S741, crucial for kinase down-regulation. The analysis has been conducted through the PyMOL Molecular Graphics System (Version 1.4.1 Schrödinger, LLC).

We analyzed in detail the structure of PIK3CA and KIT proteins and the potential influence of I391M and D737N alterations, respectively. PIK3CA^I391^ is located in the C2 domain, responsible for phospholipid membrane binding. In particular, I391 is the only residue that works as a loop between the β-sandwich, which is the core structure of the C2 domain, and an α-helix (Figure 5). The KIT^D737N^ mutation is located in the cytoplasmic portion which includes the kinase domain. In Figure 6, D737 residue is shown in green and it could be observed that it is in a loop region near to catalytic tyrosine and serine residues, in particular to S741, whose phosphorylation by PKC/PRKCA is important for the kinase down-regulation. It could be argued that the substitution of Asp737 with an Asn residue could lead to a topological change due to their different polarity.

**Figure 7:**
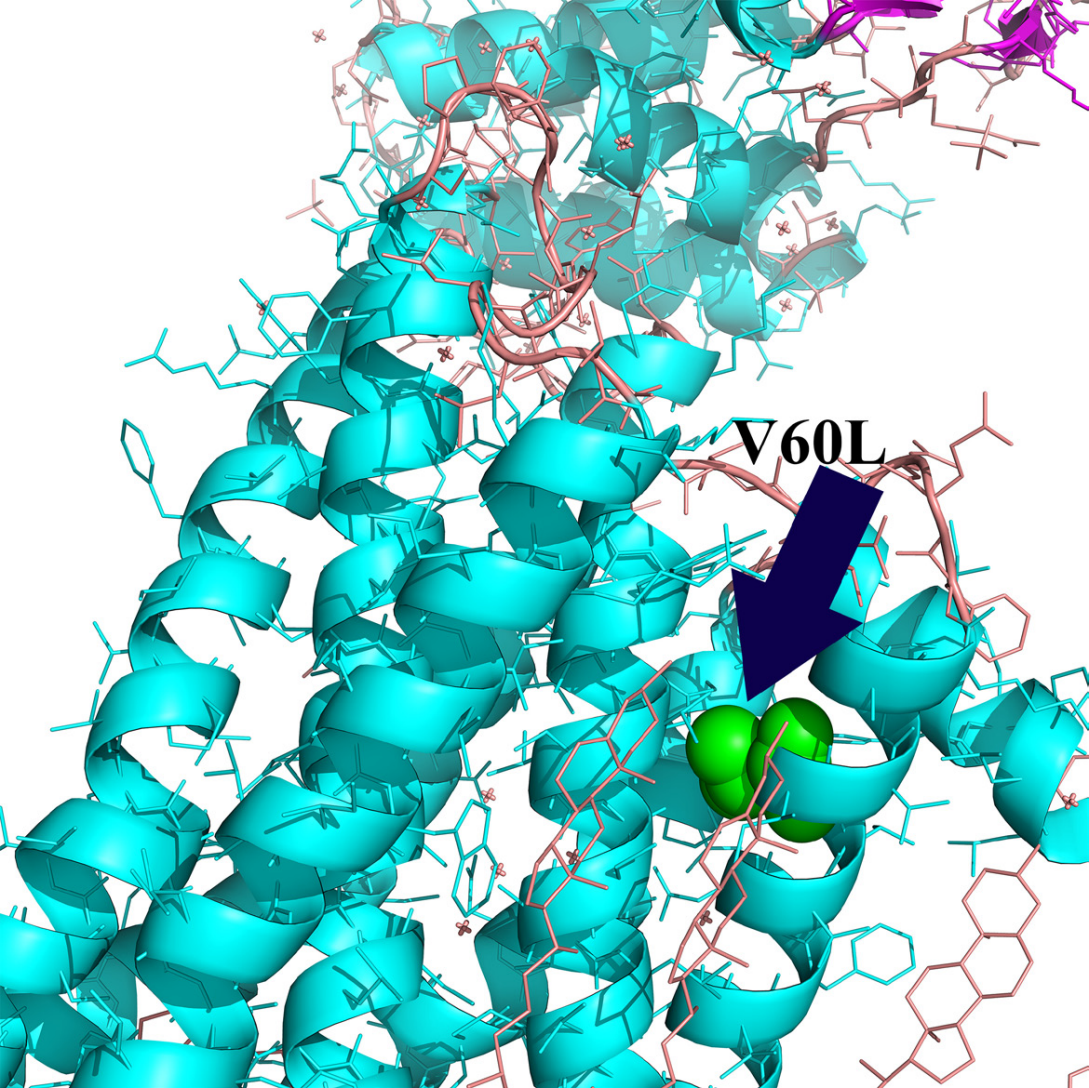
MC1R structural analysis Since there is no crystallographic data on the entire MC1R protein, we aligned the sequences of β-adrenergic receptor structure and the MC1R consensus sequence in order to identify the position of the variant (residue in green) in the β-adrenergic receptor sequence (V54). The residue MC1R^V60^ is located in the Helix I of the seven transmembrane domain structure of the receptor. The analysis has been conducted through the PyMOL Molecular Graphics System (Version 1.4.1 Schrödinger, LLC).

Regarding the potential structural implication of the MC1R^V60L^ variant, there are no crystallographic data on the entire MC1R protein. Therefore, taking into account the approach of Ibarrola-Villava et al [17], we aligned the sequences of β-adrenergic receptor structure and the MC1R consensus sequence in order to identify the position of the MC1R V60 residue in the β-adrenergic receptor sequence (V54). We observed that this residue is located in the Helix I of the seven transmembrane domain structure of the receptor (Figure 7). Moreover, the change to a leucine residue leads to a lower Kyte and Doolittle hydrophobicity score (data not shown). These observations seemed to suggest a possible implication of this variant in the correct folding of Helix I of MC1R.

## DISCUSSION

The identification of biomarkers able to predict therapeutic response is an area of intense research due to the lack of an established algorithm which defines what and when BRAF target therapy has to be placed in the treatment of MM patients. To date in absence of sequential prospective studies, the choice of the correct agent is mostly guided empirically by clinical features such as bulk of disease and its evolutional speed as well as patient characteristics such as performance status, age and presence of comorbidities. All together these issues explain how urgent is to explore new pathways to search for biomarkers which could determine the effective treatment sequence in the single patient. Furthermore, the only BRAF mutation is insufficient to predict the time of response to BRAF. Disease progression often happens within 6–8 months after the beginning of therapy due to the recovery of MAPK signaling or activation of alternative signaling pathways. Therefore, a multigene diagnostic approach, starting from a limited amount of DNA, has become mandatory in routine clinical practice for the better comprehension of the molecular mechanisms underlying this very small range of response time. However, the low amount of material available in most cases does not allow a comprehensive molecular characterization by conventional techniques. In the present study, we showed that targeted NGS using the Ion Torrent technology provides simultaneously information about multiple genes starting from a very limited amount of DNA and in a short time. Furthermore, we implemented a custom gene panel able to evidence possible other prognostic factors associated to BRAF inhibitors response. All our analyzed samples showed at least one mutation/SNP among the 11 melanoma-related genes which constituted our panel. We demonstrated a higher accuracy of NGS methods in the detection of BRAF and NRAS mutations (in 68% and 24% of samples, respectively) than Sanger and ARMS analysis. This was probably due to the low proportion of mutated alleles not detectable by traditional sequencing methods. Apart from the mutations known to be associated with metastatic melanoma, we have more frequently identified two potential pathologic mutations, PIK3CA^I391M^ and KIT^D737N^, and three polymorphisms, MITF^S473A^, CTLA4^T17A^ and MC1R^V60L^. The alterations in PIK3CA, KIT, CTLA4 and MC1R were already known. Moreover, for the first time, we have demonstrated in a subset of BRAF^V600^ patients treated with BRAF inhibitors that those without these alterations had a higher median PFS. Usually these genes resulted low mutated in cutaneous melanomas, since the entire coding regions of these genes have not been well investigated. In fact, in melanomas, hotspot regions or amplification of KIT, PIK3CA, MITF and CTLA4 are often analyzed [18–22]. MC1R^V60L^ is often associated with pigmentary phenotypes and risk of malignant melanoma [23–24]. Thus this study represents the first comprehensive analysis of these genes in metastatic melanoma patients in relation to the treatment with BRAF inhibitors.

The KIT gene encodes for a receptor tyrosine kinase that can activate multiple downstream signalling pathways, including the RAS-RAF-MEK-ERK and PI3K-AKT axis. The role of KIT in the development of malignant melanoma has been largely discussed. Authors have demonstrated in melanomas wild type for NRAS and BRAF, that activating constitutively KIT mutations may be functionally equivalent to constitutive activation of the RAS-RAF-MEK-ERK pathway due to NRAS or BRAF [25]. KIT somatic mutations are present mainly in mucosal melanoma, but they can also be found in cutaneous melanomas [26, 27]. The most frequently investigated KIT mutations in melanoma concern the exons 11 and 13 that resulted partially responsive to KIT inhibitors [28]. In fact, melanoma patients with a KIT mutation affecting a recurrent hot spot, such as the L576P or K642E mutation, have better clinical outcomes than those without a hotspot mutation [29]. Mutations in the gene encoding the catalytic subunit of PI3K, PIK3CA, occur at very low frequencies (<5%) in melanoma [30], although are very frequent in some human cancers leading to constitutive AKT activation [31]. The PI3K-AKT pathway is an important regulator of cell growth, proliferation, differentiation, metabolism, motility, and survival. Moreover, it is highly active in most metastatic melanomas, and its inhibition, together with the RAS-RAF-MEK-ERK pathway block, can lead to suppression of melanoma growth. The most frequent melanoma-associated genetic events in PI3K-AKT signaling pathway are inactivating mutations (often related only to exons 9 and 20 of PIK3CA) or PTEN loss of expression or mutations and activating NRAS mutations [32]. However, it has been demonstrated that PIK3CA mutations may contribute to RAF inhibitor resistance [33]. The MAPK pathway regulation seems to be strongly linked to MITF expression and function [34]. In fact it has been shown that the hyper-activation of RAS-RAF-MEK-ERK signalling causes a significant reduction in MITF levels and therefore melanocytes and melanoma cells proliferation [35]. Amplification of MITF is observed in 10% of primary and in 15–20% of metastatic melanomas and so it may be considered an oncogene in melanoma. In addition, MITF mutations, such as the recurrent E318K, can predispose to melanoma development [36, 37]. Regarding the function of MITF in therapy response, growing evidence has highlighted a role in innate and acquired resistance to MAPK pathway inhibitors. MITF up-regulation, related to increased tolerance and resistance to MAPK pathway inhibitors, is supported by the fact that some patients relapse with a MITF gene amplification [33]. However, this up-regulation correlates with an increased lymphocyte T CD8+ infiltration [38], and it could be advantageous for an immunotherapy approach. In our study, we demonstrated that MITF^S473A^ in association to CTLA4^T17A^ showed a higher sensitivity and specificity (AUC 0.7) in predicting shorter PFS of the considered patients, as reported for the single alterations in CTLA4, PIK3CA and KIT.

The CTLA4, a member of the CD28 superfamily, is not constitutively expressed on T cells but it is induced after CD28 binding and activation. It is responsible for the attenuation of immune response by binding to ligands (B7-1 and B7-2) expressed on the surface of antigen presenting cells. CTLA-4 gene polymorphisms have been associated with numerous autoimmune conditions, including diabetes and inflammatory bowel disease [39]. Among the numerous CTLA4 polymorphisms, the recurrent T17A substitution (rs231775) can be considered one cause of an altered endoplasmic reticulum trafficking and/or processing of CTLA4 leading to its differential expression on the cell surface [40]. We showed a higher hydrophobicity and propensity to form α-helix for CTLA4^T17A^ peptide than the wild-type one. Moreover, this non-synonymous polymorphism seems to be associated with the anti-CTLA4 therapy response [41]. There is evidence that the BRAF inhibition exerts an influence on immunological landscape of cutaneous melanoma [42–44]. In fact, BRAF inhibitors treatment is associated with decreased production of the immunosuppressive factor IL10 and enhanced expression of tumor-specific antigens [45] and so BRAF inhibitors might induce T-cell infiltration before treatment with immunotherapy. In our study, we found that patients without the CTLA4^T17A^ variant had a higher median PFS after BRAF inhibitors treatment. In future studies, it would be interesting to see whether this variant is also associated with lower immune response due to BRAF inhibitors treatment.

MC1R is a central control point in skin and hair pigmentation. Moreover, inherited variation in the MC1R gene is considered a genetic marker for moderately increased risk of melanoma [23]. In a recent study, MC1R variants were correlated with tumor characteristics suggesting that inherited variation in MC1R may play a role in determining the anatomic site of melanomas and may differ with respect to skin pigmentation phenotype [46]. The red hair colour phenotype is due to the production of more pheomelanin than eumelanin, which is a result of an altered function of MC1R. High-penetrance R alleles are the variants D84E, R151C, R160W and D294H, strongly associated with red hair and fair skin phenotypes; while the variants V60L, V92M, and R163Q are low penetrance r alleles. These three variants are often predicted neutral and tolerant by most tools [47] as demonstrated also by our group for V60L. However, for the first time, we reported a significant association of MC1R^V60L^ to melanoma progression.

In conclusion, the application of an Ampliseq Custom Panel on the Ion Torrent Personal Genome Machine for a routine clinical practice needs validation in a larger series of cases. However, these preliminary results evidenced a higher sensitivity and specificity in detecting a wide range of genetic alterations than traditional sequencing methods. Therefore a NGS approach with a custom panel may provide crucial and new information to evaluate tumor-specific therapeutic susceptibility and individual prognosis to improve the care of metastatic melanoma patients.

## MATERIALS AND METHODS

### Ethics statement

The study was approved by the local Ethics Committee of the Istituto Tumori “Giovanni Paolo II” of Bari (prot. no. 515/EC of May 12, 2015) and was conducted in accordance with the international standards of good clinical practice. All patients signed an informed consent. Moreover, all samples and medical data used in this study have been anonymized.

### Patient information

A series of 25 cutaneous melanoma patients (12 female and 13 male) with histologically confirmed stage IV melanoma who started a first line treatment at the Oncology Department of the IRCCS Istituto Tumori ‘Giovanni Paolo II’ in Bari (Italy) were retrospectively evaluated. Clinical features of patients are listened in Table 2. The period of enrollment was from July 2011 to February 2013.

**Table 2:** Baseline clinical features of all 25 cutaneous metastatic melanoma patients

	*Overall*	*BRAF inhibitors treated patients*	*Patient not treated with target therapy*
***Patients***	25	17	8
***Median age***	58 (30-82)	58 (36-82)	58 (30-71)
Female	11	8	3
Male	14	9	5
***Site of primary melanoma***			
skin	25	14	8
unknown	0	3	0
Stage IV*			
M1a	6	3	3
M1b	4	1	3
M1c	15	13	2
***Brain involvement***	3	2	1
***ECOG status***			
0	18	12	6
1	5	5	0
2	2	0	2

* According to the AJCC melanoma staging system

Seventeen from this population of MM patients were treated with BRAF inhibitors according to the presence of BRAF V600 mutation (V600E in 14 patients and V600K in 3 patients). Target therapy consisted of Vemurafenib (15 patients) and Dabrafenib (2 patients) at the standard dose of 960 mg and 150 mg respectively twice daily until progression. This subset of patients was included in our analysis. Patients were selected if they had measurable lesions; adequate renal, hepatic and bone marrow functions; an Eastern Cooperative Oncology Group (ECOG) performance status ≤ 2; a life expectancy of more than 12 weeks and it did not need dose reduction or withholdings of doses of BRAF inhibitors for related toxicities.

Patients were underwent to clinical and laboratory exams every 4 weeks and radiological evaluation with tumor assessments at baseline and then approximately every 12 weeks in order to evaluate therapeutic effectiveness. Response Evaluation Criteria In Solid Tumors (RECIST) version 1.1 was used for efficacy assessment [48]. We assessed the best response during BRAF inhibitors as complete response (CR), partial response (PR), stable disease lasting for at least 12 weeks (SD) and progressive disease (PD). We also measured PFS, defined as the length of time (in months) from the start of the treatment until disease progression.

As best response we assessed 2 CR, 10 PR and 5 PD. Median follow-up was 8 months and at the time of the final observation (January 2015) the median value of PFS was 7 months (range 1-36+ months). At the final observation, all patients but two had progressed after BRAF inhibitor treatment.

### DNA preparation

DNA was isolated from FFPE samples, after deparaffinization in xylene of 10 mm thick paraffin sections (two sections/each sample), using the QIAamp DNA FFPE Tissue Kit (Qiagen) according to the manufacturer's instructions.

### Ion Torrent PGM library preparation and sequencing

The custom panel, developed with Ion Ampliseq Designer tool (www.ampliseq.com), was used to analyze the entire coding region of 11 genes (BRAF, NRAS, PTEN, MITF, CDK4, MGMT, CTLA4, PIK3CA, MC1R, KIT, RB1) with a coverage of 93.85%. We verified that the gene regions lost in the panel were not the exon regions of our interest. These genes are crucial because they are involved in melanoma carcinogenesis and treatment response. The panel size was 39.08Kb, contained 303 amplicons, and for the analysis an input of 20ng of FFPE DNA (10 nanograms/each primer pool) was required. Ion Torrent adapter-ligated libraries were made following the manufacturer's protocol for the Ion AmpliSeq Library Kit 2.0 (Life Technologies). AMPure beads (Beckman Coulter) were used to purify the resulting libraries. To determine the concentration and size of the libraries, we used two methods: the Agilent 2100 BioAnalyzer (Agilent Technologies) with the Agilent BioAnalyzer DNA High-Sensitivity kit (Agilent Technologies) and the Ion Library Quantitation kit (Life technologies).

Sample emulsion PCR and enrichment were performed using the Ion PGM Template OT2 200 Kit, according to the manufacturer's instructions. The Ion 314 and 316 Chip Kit v2 (Life Technologies) were used for sequencing on the Ion Torrent PGM barcoded samples. The Ion PGM 200 Sequencing Kit was used for sequencing reactions (Life Technologies).

### Variant calling and experimental validation

Data from the PGM runs were processed initially using the Ion Torrent platform-specific pipeline software Torrent Suite to generate sequence reads, trim adapter sequences, filter, and remove poor signal-profile reads. Initial variant calling from the Ion AmpliSeq sequencing data was generated using Torrent Suite Software v4.2 with a plug-in “*variant caller v4.2*” program. In order to eliminate errors in base calling, Somatic-High Stringency parameters setting was used to generate the final variant calling. Filtered variants were annotated using the Ion Reporter software v4.2 (Life Technologies). Mutations were visually examined using Integrative Genomics Viewer (IGV) software (http//www.broadinstitute.org/igv).

BRAF^V600^ and NRAS^Q61^ missense mutations were confirmed by Sanger's sequencing and the Real-Time PCR ARMS method (Qiagen) respectively.

### Prediction tools analysis

Six computational tools were used to predict the effect of aminoacid substitutions on CTLA4, MITF, PIK3CA, KIT and MC1R. In detail, SIFT [49], Polyphen-2 [50], PROVEAN [51], CONDEL [52], SNPS&GO [53] and Panther [54] were used to perform predictions. They are based on phylogenetic and structural information, which allow to obtain a score indicating how aminoacid substitutions could change the protein structure.

### Protein structural analysis

To analyze CTLA4^T17A^ variation, we started performing prediction through SignalIP 3.0 (http://www.cbs.dtu.dk/services/SignalP-3.0/) [55]. To clarify its influence in secondary structure, we analyzed both hydropathicity in the Kyte and Doolittle scale [56] and α-helix propensity in the Chou and Fasman scale [57] (http://web.expasy.org/protscale/) [58]. The NetPhos 2.0 (http://www.cbs.dtu.dk/services/NetPhos/) was the bioinformatic tool used to predict phosphorylation sites of MITF [59].

The PyMOL Molecular Graphics System (Version 1.4.1 Schrödinger, LLC) was used to tridimensionally evaluate PIK3CA, KIT and MC1R structure. In detail, PDB IDs for PIK3CA was 3HIZ [60]. KIT structure was downloaded by Protein Model Portal (http://www.proteinmodelportal.org/) [61] and provided by SwissModel (http://swissmodel.expasy.org/) [62]. KIT ID was 4hvsA. To analyze the structure of MC1R, the recent β-adrenergic receptor structure (PDB ID: 2RH1) has been taken into account [63]. The structural evaluation of the V60L variants was performed firstly aligning through BLASTP [64] β-adrenergic G protein-coupled receptor to the consensus sequence of MC1R.

### Statistical analysis

Statistical analyses were carried out through R v3.2.0. The Chi-square test was performed through the chisq.test command. The univariate and multivariate analyses were carried out fitting generalized linear model through the glm() function of R package “MASS”. The “survival” R-package was used to fulfill Cox regression analysis taking into account PFS. PFS probability was computed by comparing the Kaplan-Meier curves through the log rank test by GraphPad Prism 5.0.1 (GraphPad Prism 5.01, San Diego, CA, USA). Results from all statistical analyses were considered to be significant at a level of *p*-values less than 0.05.

Receiver operating characteristic (ROC) curves and the area under the ROC curve (AUC) were used to assess the sensitivity and specificity of the most frequent alterations and their combination with respect to clinical response to BRAF inhibitors treatment (partial response/stable disease or progression established through RECIST criteria). The ROC analysis was performed through R-package “pROC” (available at http://expasy.org/tools/pROC/ under the GNU General Public License).

## SUPPLEMENTARY TABLE




