# Supplementary material for: The next generation of metastatic melanoma: uncovering the genetic variants for anti-BRAF therapy response
Source: Oncotarget. 2016 Feb 3;7(18):25135–49. doi: 10.18632/oncotarget.7175 (PMC5041894; doi:10.18632/oncotarget.7175)
Supplement: Supplementary file 1 [file oncotarget-07-25135-s001.pdf]

SUPPLEMENTARY TABLE

Supplementary Table 1: The table contains all variants identified in the 25 metastatic melanoma patients studied with a custom gene panel in NGS

See Supplementary File 1
