# Supplementary material for: The next generation of metastatic melanoma: uncovering the genetic variants for anti-BRAF therapy response
Source: Oncotarget. 2016 Feb 3;7(18):25135–49. doi: 10.18632/oncotarget.7175 (PMC5041894; doi:10.18632/oncotarget.7175)
Supplement: Supplementary file 2 [file oncotarget-07-25135-s002.doc]

|  |  |  |  |  |  |  |  |  |  |  |  |
| --- | --- | --- | --- | --- | --- | --- | --- | --- | --- | --- | --- |
| ***pts*** | ***NRAS*** | ***CTLA4*** | ***MITF*** | ***PIK3CA*** | ***KIT*** | ***BRAF*** | ***MGMT*** | ***PTEN*** | ***CDK4*** | ***RB1*** | ***MC1R*** |
| **1** | p.Gly12Asp| | p.Thr17Ala | p.Ser473Ala | p.Ile391Met | p.Asp737Asn | p.Val600Lys |  | p.Lys344Arg |  |  | p.Val92Met |
|  |  |  |  | p.Glu707Lys |  | p.Tyr85Cys |  | p.Pro367Ser |  |  | p.Arg151Cys |
|  |  |  |  |  |  |  |  |  |  |  | *c.942A>G* |
| **2** | p.Gly138Glu | p.Thr17Ala | p.Ser473Ala | p.Asp152Asn | p.Pro27Leu | p.Thr753Ser | p.His116Tyr | p.Thr2Ile |  | p.Arg556Leu | p.Ala105Val |
|  | *c.192C>T* | p.Trp20Leu | *c.1404C>A* | p.Trp424Ter | p.Ser30Gly | p.Gly315Ter | p.Ser124Leu | p.Cys83Tyr |  | p.Gly865Arg | p.Arg151Cys |
|  |  | *c.468G>T* |  | p.Leu653Ile | p.Ile539Ser | p.Gln153Glu |  | p.Phe195Leu |  | p.Arg73Gly |  |
|  |  |  |  | p.Ala869Val | p.Val211Met | p.Thr753Ser |  | *c.882T>C* |  | p.Gln354Arg |  |
|  |  |  |  | p.Asn996Ser | p.Asp737Asn | p.Trp619Leu |  | *c.513G>A* |  | p.Val419Met |  |
|  |  |  |  | *c.171C>A* | p.Asp765Asn | p.Val600Glu |  | *c.558G>A* |  | p.Thr922Asn |  |
|  |  |  |  | *c.3072G>A* | *c.1620G>A* | p.Ile371Thr |  |  |  | p.Glu927Lys |  |
|  |  |  |  |  | *c.541C>A* | p.Arg347Gln |  |  |  | *c.1389A>G* |  |
|  |  |  |  |  | *c.816G>A* | p.Glu96Lys |  |  |  | *c.2757C>T* |  |
|  |  |  |  |  |  | *c.1512A>G* |  |  |  |  |  |
|  |  |  |  |  |  | *c.495G>A* |  |  |  |  |  |
|  |  |  |  |  |  | *c.1035C>A* |  |  |  |  |  |
|  |  |  |  |  |  | *c.1821C>T* |  |  |  |  |  |
| **3** | p.Val109Ile |  | p.Ser473Ala | p.Glu707Lys | p.Asp737Asn | p.Gln153Glu | p.Leu115Phe |  | p.Tyr165Cys | p.Phe57Ile | p.Arg151Cys |
|  |  |  |  | p.Val71Ile | p.Arg177Cys | p.Glu96Lys | p.Gly153fs |  |  | p.Thr922Asn |
|  |  |  |  | p.Arg93Trp | p.Ile748Leu | p.Tyr85Cys | *c.252C>T* |  |  | *c.1153T>C* |  |
|  |  |  |  | p.Val952Gly | p.Pro754Ser | p.Thr753Ser |  |  |  |  |  |
|  |  |  |  |  | p.Asp759Asn | p.Val600Glu |  |  |  |  |  |
|  |  |  |  |  |  | p.Ser536Asn |  |  |  |  |  |
|  |  |  |  |  |  | p.Arg347Gln |  |  |  |  |  |
|  |  |  |  |  |  | *c.1929A>G* |  |  |  |  |  |
| **4** | p.Ala134Asp |  | p.Ser473Ala | p.Ile391Met | p.Asp737Asn | p.Arg347Gln | p.Leu115Phe |  | p.Gly15Asp | *c.328C>T* | p.Val60Leu |
|  | *c.423C>T* |  | *c.1404C>A* | p.Glu707Lys | p.Ile539Ser | p.Ile342Thr | p.Ile174Val |  |  |  | p.Leu202Phe |
|  |  |  |  | p.Gly828Ser | p.Ala837Val | p.Phe290Leu |  |  |  |  | p.Arg160Trp |
|  |  |  |  | *c.2088G>A* |  | p.Ala34Val |  |  |  |  |  |
|  |  |  |  |  |  | p.Val600Glu |  |  |  |  |  |
|  |  |  |  |  |  | p.Tyr85Cys |  |  |  |  |  |
|  |  |  |  |  |  | *c.1929A>G* |  |  |  |  |  |
| **5** |  |  |  |  | p.Met541Leu | p.Val600Glu | p.Leu115Phe | p.Arg84Ter |  |  |  |
|  |  |  |  |  | *c.2586G>C* | *c.1929A>G* |  |  |  |  |  |
| **6** | p.Gln61Leu | p.Thr17Ala | p.Asn203Ile | p.Ile391Met | p.Asp737Asn | p.Glu96Lys |  |  |  |  | p.Val60Leu |
|  |  |  |  |  |  | p.Lys88Thr |  |  |  |  |  |
|  |  |  |  |  |  | p.Tyr85Cys |  |  |  |  |  |
| **7** |  | p.Thr17Ala | p.Ser473Ala | p.Ile391Met | p.Arg49His | p.Ala320Thr | p.Leu115Phe | p.Asp297Asn |  | p.Ala628Gly | p.Ala161Thr |
|  |  |  | *c.1404C>A* | p.Glu707Lys | p.Val213Leu | p.Gln153Glu | *c.252C>T* | *c.480C>T* |  |  |  |
|  |  |  |  | *c.1504C>A* | p.Pro341His | p.Ser110Phe |  | *c.516G>A* |  |  |  |
|  |  |  |  |  | p.Thr391Ile | p.Tyr85Cys |  |  |  |  |  |
|  |  |  |  |  | p.Gly498Ser | *c.1035C>A* |  |  |  |  |  |
|  |  |  |  |  | p.Asp737Asn |  |  |  |  |  |  |
|  |  |  |  |  | p.Ile748Leu |  |  |  |  |  |  |
|  |  |  |  |  | *c.541C>A* |  |  |  |  |  |  |
|  |  |  |  |  | *c.1023C>A* |  |  |  |  |  |  |
|  |  |  |  |  | *c.1029T>A* |  |  |  |  |  |  |
|  |  |  |  |  | *c.1491G>A* |  |  |  |  |  |  |
| **8** |  |  |  |  | p.Asp737Asn | p.Lys88Thr | p.Leu115Phe |  |  |  |  |
|  |  |  |  |  | p.Pro832Ser |  | *c.252C>T* |  |  |  |  |
|  |  |  |  |  | *c.297C>T* |  |  |  |  |  |  |
| **9** | *c.522T>C* | p.Thr17Ala |  | p.Ile391Met | p.Asp737Asn | p.Val600Glu | p.Ile174Val |  |  | p.Gln899Lys | p.Val60Leu |
|  |  |  |  | p.Gly685Asp |  |  | p.Lys209Arg |  |  |  | p.Arg163Ter |
|  |  |  |  |  |  |  |  |  |  |  | *c.942A>G* |
| **10** |  | p.Thr17Ala | p.Ser473Ala | p.Met441Ile | p.Met541Leu | p.Val600Glu |  | p.Thr350Ile |  |  | p.Arg160Trp |
|  |  |  |  |  |  | p.Ile342Thr |  | p.Ser355Leu |  |  |  |
|  |  |  |  |  |  | p.Lys88Thr |  |  |  |  |  |
|  |  |  |  |  |  | p.Tyr85Asn |  |  |  |  |  |
| **11** |  | p.Thr17Ala | p.Ser473Ala | p.Glu707Lys |  | p.Ile342Thr | p.Ile174Val |  |  |  | p.Val60Leu |
|  |  |  | *c.1404C>A* |  |  | *c.1929A>G* | p.Lys209Arg |  |  |  |  |
| **12** |  | p.Thr17Ala | p.Asn188Tyr | p.Ala314Thr | p.Asn80Lys | p.Thr753Ser | p.Lys209Arg |  |  | p.Arg73Gly | p.Asp294His |
|  |  | *c.468G>T* | p.Met245Ile | p.Ser482Gly | p.Ala599Asp | p.Val600Lys | *c.252C>T* |  |  | p.Ile101Val |  |
|  |  |  | p.Lys307Glu | p.Arg852Ter | *c.541C>A* | *c.1035C>A* |  |  |  | p.Thr922Asn |  |
|  |  |  | p.Ser473Ala | *c.465C>T* | *c.1638A>G* |  |  |  |  |  |  |
|  |  |  | *c.1404C>A* | *c.942T>C* |  |  |  |  |  |  |  |
| **13** | p.Gln61Lys | p.Thr17Ala | p.Asn203Ile | p.Val125Ala | *c.2403T>C* | *c.1929A>G* |  | p.Ile4Thr |  |  | p.Val60Leu |
|  |  |  | p.Ser473Ala | p.Ile391Met |  |  |  | *c.15C>T* |  |  | p.Arg160Trp |
|  |  |  |  | *c.942T>C* |  |  |  |  |  |  |  |
| **14** |  |  | p.Ser473Ala |  | p.Thr801Ile | p.Thr753Ser | *c.252C>T* |  |  |  | p.Val60Leu |
|  |  |  |  |  | *c.541C>A* | p.Val600Glu | *c.621G>A* |  |  |  |  |
|  |  |  |  |  |  | p.Arg347Gln |  |  |  |  |  |
|  |  |  |  |  |  | p.Ile342Thr |  |  |  |  |  |
|  |  |  |  |  |  | *c.1035C>A* |  |  |  |  |  |
| **15** | p.Gln61Arg | p.Pro205Ser | p.Ser473Ala | p.Arg398Ser | p.Met541Leu | p.Val600Glu | p.Lys209Arg | p.Arg161Lys |  | p.Ser485Phe | p.Asn68Lys |
|  |  | p.Pro206Thr | *c.801C>T* | p.Leu653Ile | *c.541C>A* | p.Pro348Leu | p.Gly220Glu | p.Arg308His |  | *c.2757C>T* | p.Arg151Cys |
|  |  | *c.489C>T* |  | *c.1915C>T* | *c.1041G>A* | p.Ile342Thr | *c.252C>T* | p.Ala328Glu |  |  | p.Arg160Trp |
|  |  |  |  | *c.1962G>A* | *c.2586G>C* | p.Tyr78Cys |  | *c.946C>T* |  |  | *c.183C>A* |
|  |  |  |  | *c.2355C>T* | *c.2790G>A* | *c.235C>T* |  |  |  |  |  |
| **16** |  | p.Thr17Ala | p.Ser473Ala | p.Ile391Met | *c.1638A>G* | p.Arg347Gln |  |  |  | p.Gly90Asp |  |
|  |  |  |  |  |  | p.Ile342Thr |  |  |  | p.Arg455Ter |  |
|  |  |  |  |  |  | *c.1929A>G* |  |  |  |  |  |
| **17** | p.Gln61Arg |  | p.Ser473Ala | p.Asp488Asn | p.Val742Met | p.Thr753Ser | p.Leu115Phe |  |  |  | p.Arg151Cys |
|  |  |  |  | p.Thr624Ile | p.Ile748Leu | p.Ile342Thr | p.Ile174Val |  |  |  |  |
|  |  |  |  | p.Cys695Tyr | *c.541C>A* | p.Lys88Thr | *c.252C>T* |  |  |  |  |
|  |  |  |  | p.Glu707Lys | *c.2394C>T* | p.Tyr85Cys |  |  |  |  |  |
|  |  |  |  |  |  | *c.564A>G* |  |  |  |  |  |
| **18** |  |  | p.Met471Lys | p.Arg4Ter | p.Asp737Asn | p.Thr753Ser |  | p.Met205Ile | p.Gly111Arg | p.Thr922Asn |  |
|  |  |  | p.Ser473Ala | p.Ile391Met | *c.541C>A* | p.Val600Glu |  |  |  | *c.328C>T* |  |
|  |  |  | *c.1404C>A* | *c.1504C>A* | *c.1023C>A* | p.Glu96Lys |  |  |  | *c.2757C>T* |  |
|  |  |  |  |  |  | p.Tyr85Cys |  |  |  |  |  |
|  |  |  |  |  |  | *c.1035C>A* |  |  |  |  |  |
| **19** | p.Val112Met |  |  | p.Met1Ile | p.Cys491Tyr | p.Val600Arg |  | p.Val158Ile |  | p.Arg455Gln |  |
|  |  |  |  | p.Ser571Phe | p.Pro573Ser | p.Pro420Leu |  |  |  | p.Ile752Thr |  |
|  |  |  |  | p.Ala775Thr | p.Asp765Asn | *c.1986G>A* |  |  |  | *c.1362C>T* |  |
|  |  |  |  | *c.1473G>A* | *c.1065C>T* | *c.1929A>G* |  |  |  | *c.2100G>A* |  |
|  |  |  |  | *c.1527C>T* |  | *c.1839G>A* |  |  |  | *c.2247T>C* |  |
|  |  |  |  | *c.2355C>T* |  | *c.1548C>T* |  |  |  |  |  |
| **20** |  | p.Thr17Ala |  |  | p.Met541Leu | p.Val600Glu |  |  |  |  |  |
|  |  |  |  |  | *c.2586G>C* | *c.1929A>G* |  |  |  |  |  |
| **21** |  |  |  | p.Glu707Lys | p.Asn652Asp | p.Val600Glu |  |  |  |  |  |
|  |  |  |  |  |  |  |  |  |  |  |  |
| **22** | p.Gln61Lys | p.Thr17Ala |  | p.Ile391Met |  |  | p.Ile174Val |  |  |  |  |
|  |  |  |  | *c.594T>C* |  |  | p.Lys209Arg |  |  |  |  |
|  |  |  |  |  |  |  |  |  |  |  |  |
| **23** |  |  |  | p.Ile391Met |  | p.Val600Glu | p.Leu115Phe |  |  | *c.2550G>A* | p.Gly43Arg |
|  |  |  |  |  |  | p.Pro376Ser | *c.252C>T* |  |  |  | p.Val60Leu |
|  |  |  |  |  |  |  |  |  |  |  | p.Arg160Trp |
| **24** |  |  |  |  |  | p.Val600Glu |  |  |  |  |  |
|  |  |  |  |  |  |  |  |  |  |  |  |
| **25** | p.Gln61Arg | p.Thr17Ala | p.Ala38Asp | p.Ile391Met | *c.541C>A* | *c.1035C>A* |  |  |  | p.Thr645Ile | p.Val60Leu |
|  |  |  | p.Ser473Ala |  |  |  |  |  |  |  |  |

Supplementary Table 1: The table contains all variants identified in the 25 metastatic melanoma patients studied with a custom gene panel in NGS.
